# The Oral Health Condition of Patients on Hemodialysis at a Tertiary Healthcare Facility in Eastern Nepal

**DOI:** 10.1155/2024/3776702

**Published:** 2024-02-12

**Authors:** Naresh Prasad Joshi, Ashish Shrestha, Tarakant Bhagat, Santosh Kumari Agrawal, Roshan Chhetri

**Affiliations:** ^1^Public Health Dentistry, Chitwan Medical College, Bharatpur, Nepal; ^2^Department of Public Health Dentistry, BP Koirala Institute of Health Sciences, Dharan, Koshi, Nepal; ^3^Department of Internal Medicine, BP Koirala Institute of Health Sciences, Dharan, Koshi, Nepal

## Abstract

**Objectives:**

This study was done to compare the oral health of chronic kidney disease (CKD) patients on hemodialysis to healthy individuals and to assess the effect of the duration of dialysis on oral health.

**Materials and Methods:**

A comparative cross-sectional study was conducted among purposively selected 54 cases undergoing dialysis and 54 apparently healthy controls. Caries experience and periodontal status were recorded under similar examination conditions and compared between cases and controls. The correlation between oral health and the duration of dialysis was assessed. A *p* value of <0.05 was considered significant.

**Results:**

The mean age of the participants was 47.09 ± 12.23 years. Median caries experience among cases and controls was two (0–26) and three (0–15), respectively (*p*=0.013). Periodontitis among the two groups was found to be significantly different with a greater number of cases showing increased severity (*p*  < 0.001). The severe form of periodontitis was associated with a greater mean duration of dialysis.

**Conclusion:**

Significant differences exist between the periodontal health of patients undergoing dialysis compared to healthy controls. Longitudinal studies are required to check the causal effect of CKD on periodontitis.

## 1. Introduction

Chronic kidney disease (CKD) is increasingly being recognized as a global public health problem [1]. It may arise from different disease pathways leading to an alteration of the function and structure of the kidney irreversibly, over months or years. It is defined as kidney damage or glomerular filtration rate (GFR) <60 mL/min/1.73 m^2^ for 3 months or more, irrespective of cause [2]. While diabetes mellitus is the most common cause of CKD, causes include hypertension, glomerulonephritis, polycystic kidney disease, and pyelonephritis among others [3, 4]. It has a high global prevalence of 11%–13% [5]. CKD patients experience a wide variety of symptoms and complications as the disease progresses. As the kidney functions become marginal, complications related to uremia such as pericarditis, pulmonary edema, neurological problems, and/or metabolic abnormality start to develop.

It is a common practice to start renal replacement therapy after Stage 5 CKD (GFR < 15mL/min/1.73 m^2^) but before renal functions cease [6]. Renal replacement therapy includes hemodialysis (HD), peritoneal dialysis, and kidney transplantation among which, HD is the most common form of intervention. It is instrumental in the survival of more than a million people throughout the world who have end-stage renal disease (ESRD) with limited or no kidney function. The primary goal of performing HD is to restore intracellular and extracellular environment similar to normal kidney function. The standard recommended frequency of dialysis is three sessions per week [7].

Nepal, one of the developing countries in the world, in 143^rd^ position on the human development index [8], has limited treatment options for CKD. Despite the logistic challenges, HD services have been offered free of cost by the Government of Nepal since 2016 [9]. These services, though not adequate, are available in all seven provinces of Nepal through various hospitals. The survival of patients with CKD has improved significantly over the past decades as HD has provided a support system for kidney functions [7]. However, it does compromise different aspects of life including physical, social, familial, and financial domains. In the long run, patients on HD become more susceptible to infections because of the suppression of the immune system [10]. These patients show not only systemic changes but are also beset with oral complications [11].

Evidence for the association of CKD with caries experience and periodontal status consists of a small body of literature represented mainly by descriptive studies [12]. The association of periodontitis to systemic diseases like coronary heart disease and stroke and a higher risk of preterm low birth-weight babies has been well-known. A bidirectional nature between periodontitis and systemic disease has also been proposed [13].

There are conflicting reports about the association of CKD with caries experience and periodontal status. Some studies have shown a significant increase in decayed missing-filled teeth (DMFT) in patients with CKD undergoing HD [14, 15]. However, others report lower lifetime caries experience in patients with CKD [16]. Peterson et al. [17] argue that increased salivary urea levels can contribute to the remineralization of dental enamel leading to lower caries experience. Although existing literature suggests significantly higher periodontal disease among CKD patients in comparison to controls, some studies have not been able to identify a significant relation between impaired kidney function and oral diseases [18, 19].

To the best of the authors' knowledge, there is no study comparing caries experience and periodontal status of CKD patients with a healthy population in Nepal. This study was conducted to assess the oral health status of CKD patients on dialysis in the form of lifetime caries experience and periodontal status and compare those findings with an apparently healthy control group matched for age and gender.

## 2. Methods

A comparative cross-sectional study was done in the dialysis ward of B P Koirala Institute of Health Sciences (BPKIHS), Dharan, Nepal from April to December 2020. BPKIHS is a tertiary health care center in the eastern part of Nepal. CKD Patients of 18 years and above undergoing dialysis were purposively selected as cases while apparently healthy individuals were taken as controls. Individuals with no known systemic diseases were operationally defined as “Apparently healthy” for the purpose of this study. Cases and controls were matched for age and gender. Individuals who were unable to undergo oral examination, those with diabetes mellitus and those who were not willing to participate in the study were excluded. Ethical approval was obtained from the Institutional Review Committee (IRC), BPKIHS (Ref. No. 201/076/77-IRC) before the commencement of the study.

This study considered a 95% confidence interval, 80% power, and difference of means and standard deviations of probing depths (PDs) of gingiva between case and control groups from a study conducted in India [20]. A Software *nMaster 2.0* (Christian Medical College, Vellore, India) was used to estimate the sample size.

### 2.1. Instruments/Questionnaire

(1) DMFT/DMFS index [21]
The criteria for identification of dental caries were:
  - Clinically visible and obvious lesion;
  - The probe tip can penetrate deep into soft-yielding material;
  - Discoloration or loss of translucency typical of undermined or demineralized enamel;
  - The probe tip in a pit or fissure catches on removal after moderate to firm pressure on insertion and when there is softness at the base of the area.
  - The “M” component included teeth missing due to caries in individuals under 30 years and teeth missing due to any reason for individuals who were 30 years or older.
  - The “F” component included teeth with permanent restoration because of caries.
(2) PD and clinical attachment loss (AL) measurement for assessing periodontal status [22, 23].
PD and clinical AL were recorded for individual teeth using a calibrated periodontal probe by full mouth charting. Centers for Disease Control and Prevention (CDC)/American Academy of Periodontology (AAP) case definition for periodontitis was used.
(3) Questionnaire: The questionnaire was prepared in English and translated using the back translation method through standard WHO guidelines by three separate experts and checked for face validity by faculties in the Department of Public Health Dentistry, BPKIHS. Name, age, gender, education, brushing frequency, use of dental floss, tobacco usage, oral health problems, and last dental visit were recorded for all participants. Duration of dialysis and oral health education after starting dialysis were asked of participants in the case group only.
Current smokers included adults who had smoked 100 cigaretes in their lifetime and who were smoking cigaretes till the time of data collection; former smokers included adults who had smoked at least 100 cigaretes in his or her lifetime but who had quit smoking at the time of interview; and never smoker consisted of adults who had never smoked, or who had smoked less than 100 cigarettes in his or her lifetime.
(4) Instruments used included WHO probe, UNC-15 color-coded probe, plane mouth mirror, and explorer.

### 2.2. Data Collection

Data collection was done by the principal investigator from September to November 2020. Written informed consent was taken from all the participants after reading the information sheet. In individuals who were unable to read and write, an information sheet was read to them in the presence of a witness and thumbprints were recorded after they agreed to participate. The majority of participants had been undergoing 3.5 hr of dialysis twice a week. Interview of the participants was done using a structured questionnaire.

The questionnaire was administered by the investigator.

Patients' complete medical history was checked before clinical examinations especially considering the need for antibiotic prophylaxis before oral manipulation. Oral health status (DMFT/DMFS index and periodontal status (PD and clinical AL)) of both cases and control was recorded under natural light using appropriate pro forma with the examiner in a standing position and participants sitting on a chair. Data were recorded and entered in the pro forma by an assistant in a standing position under the direct supervision of the examiner. Cases were examined while they were waiting for their HD session. Caries detection was done using a mouth mirror and WHO probe while periodontal status was recorded using a mouth mirror and color-coded UNC-15 probe. Standard infection prevention protocols were observed during clinical examinations.

People found to be having oral health problems were informed about it and referred to the College of Dental Surgery, BPKIHS for treatment. Oral health education was provided to all participants regarding oral hygiene maintenance. Dialysis patients were informed to visit the dental hospital on the next day after dialysis.

### 2.3. Data Management and Statistical Analysis

Data were entered in Microsoft Excel 2016 and exported to the statistical package for the social sciences (SPSS) software version 11.5 for statistical analysis.

Mean, standard deviation, median, interquartile range, and proportion were calculated with graphical and tabulated presentations to illustrate age, gender, literacy, brushing frequency, use of dental floss, tobacco usage, self-reported oral health problems, last dental visit, duration of dialysis, oral health education after starting dialysis, DMFT/DMFS, and periodontitis.

Mann–Whitney *U* test was used to compare means of dental caries experience between cases and controls.

The *χ*^2^ test was used to check the association between periodontitis between cases and controls.

Spearman correlation was used to compare the duration of dialysis and caries experience while the Kruskal–Wallis test was used to compare the mean duration of dialysis with periodontitis. A *p* value of <0.05 was considered statistically significant.

## 3. Results

### 3.1. Participant Demographic

There were 108 participants in this study out of which 54 were patients of CKD undergoing dialysis at BPKIHS and 54 were apparently healthy individuals matched for age (±5 years) and gender with the cases. There were 52 (48.14%) males and 56 (51.86%) females. The mean age (SD) of the participants was 47.09 (12.23) years.

Out of 108 participants, 87 of the participants (80.5%) had received some form of education. More individuals in the case group were illiterate (24.1%) as compared to controls (14.8%).

The proportion of individuals brushing their teeth once a day was equal in the cases and control group (50%). Among cases, 12 individuals (22.2%) responded that they only brushed their teeth sometimes (infrequently). A significantly smaller number of cases brushed their teeth at least once a day (*p*=0.036). The use of dental floss was very uncommon in both groups with only two participants in each group reporting its use. Interestingly, the majority (61.1%) of participants were unaware of dental floss. Greater than two-thirds (72.2%) of cases had not visited a dentist ever in their lifetime. On the contrary, more than two-thirds (68.5%) of the individuals in the control had visited a dentist at least once in their life. The pattern of dental visits was also seen to be significantly lower among cases of CKD undergoing dialysis (*p*=<0.001).

There were no current smokers in the cases. However, 19 (35.2%) participants were previous smokers. Difference in tobacco usage in the two groups was also found to be statistically significant (*p*=<0.001) with a greater number of cases being smoker at some point in their life (Table 1).

### 3.2. Oral Health Education to Hemodialysis Patients

The median duration of HD was 23.5 months (interquartile range 30). Only one participant had received education regarding oral health after their dialysis had begun.

### 3.3. Reliability

The intraclass correlation coefficient for intraexaminer reliability of a single examiner was found to be 0.98 (excellent agreement) [24] for DMFT measure and *κ* value of 0.89 (almost perfect agreement) [25] for periodontal status when repeat examinations were conducted among 25 individuals during the study.

### 3.4. Comparison of Oral Health Status among Cases and Controls

Statistically significant differences were observed in decayed teeth (DT, *p*=0.013), filled teeth (FT, *p*=<0.001), and DMFT (*p*=0.013). Similarly, decayed surface (DS, *p*=0.001) and filled surface (FS, *p*=<0.001) between cases and controls also showed significant differences with DT, FT, DMFT, DS, and FS more in the control group. Interestingly, the difference in DMFS between the two groups was not statistically significant. The proportion of individuals with moderate and severe periodontitis was more in the case group and the difference was found to be statistically significant (p < 0.001; Table 2).

### 3.5. Self-Reported Oral Health Problems among Cases and Controls

**Figure 1:**
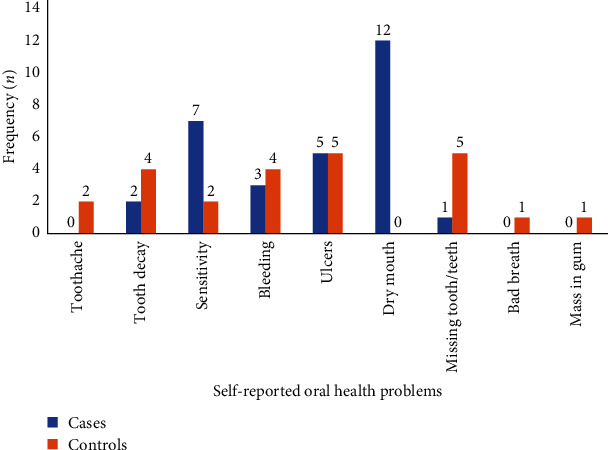
Self-reported oral health problems from BPKIHS, September–November, 2020 (*n* = 104). The other participants responded by saying they had no oral health problems.

The cases in dialysis (55.5%) had more self-reported oral problems as compared to controls (44.5%). The most commonly self-reported oral health problem among cases was found to be dry mouth followed by tooth sensitivity, oral ulcers, and gum bleeding. In the control group, the more commonly reported problem was missing teeth followed by oral ulcers and gum bleeding (Figure 1).

### 3.6. Oral Health Status according to Duration of Dialysis

The cases in our study had been on dialysis from 3 to 117 months with a median of 23.5 months.

**Figure 2:**
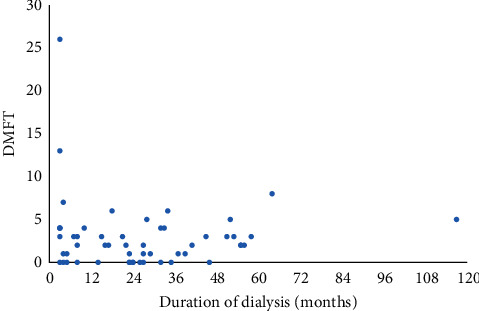
Scatter plot of DMFT versus duration of dialysis in months from BPKIHS, September–November, 2020 (*n* = 52).

Only a weak positive correlation was seen between the duration of dialysis (in months) and the caries experience of the cases (*r* = 0.1, *p*=0.982; Figure 2).

**Figure 3:**
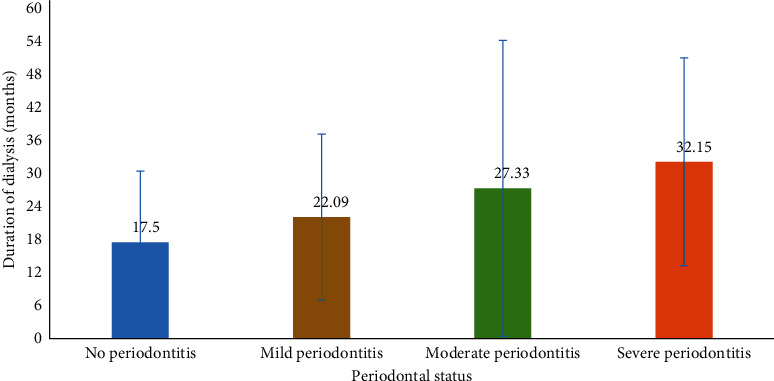
Bar diagram showing the mean duration of dialysis of cases (in months) according to the different periodontal status from BPKIHS, September–November, 2020 (*n* = 52).

On the other hand, periodontal status was seen to worsen according to the length of dialysis. However, upon analysis using the Kruskal–Wallis test, the difference was not found to be statistically significant (*p*=0.51, Figure 3).

## 4. Discussion

In our study, the oral health of CKD cases undergoing dialysis was compared in the form of DMFT and periodontitis to that of apparently healthy controls. Periodontitis was present in significantly higher number of cases. Moreover, the severity of periodontitis was also greater. On the contrary, mean DMFT score was significantly greater among the controls. We also compared caries experience and periodontitis in CKD cases according to the duration of dialysis. Worse periodontal health was seen with increased mean duration of dialysis but the difference was not statistically significant.

The mean age of the cases was 47.41 years which is similar to the mean age of patients under dialysis in a health center in central Nepal [26]. A similar mean age of 572,029 cases of ESRD has been reported in India [27]. There were 26 male and 28 female participants in each group. Female gender has been associated with better patient survival among patients undergoing dialysis in a previous study [28].

Lack of education has been implicated in the occurrence of CKD in a previous study [29]. Though more participants among the controls had secondary or higher education, the difference was not statistically significant in our study.

Our study showed significantly higher periodontitis in cases and more severe forms. Case definition of CDC/AAP based on measurement from four interproximal sites per tooth was used to define a case of periodontitis in this study. Similar case definitions have been used by a few studies lately [30–32]. Indices like community periodontal index (CPI) and community periodontal index of treatment needs (CPITN) and parameters like clinical AL have been used in different studies by different authors. However concerns have been raised regarding limitations posed by CPI and LOA in the assessment of periodontal condition, so elaborative full mouth charting of clinical AL and PD has been done in our study [33].

Many reasons have been proposed to explain the higher prevalence of periodontitis in CKD cases undergoing dialysis. First, these patients are in a state of uremic syndrome, which is associated with defects in lymphocyte and monocyte dysfunction [34]. This uremia has been implicated in increased gingival and periodontal inflammation. Second, in addition to uremia, confounding diseases like diabetes mellitus can be the putative factor especially when considering it is the most common cause of CKD [35]. Third, hyperparathyroidism caused due to secondary alteration in calcium hemostasis in kidney disease has also been suggested as a possible reason for possible alveolar bone loss in HD populations [36]. Final, decreased utilization of dental services in cases has been reported by studies [37, 38]. Probably, intense psychological demands and lack of personal time faced by CKD patients because of the need for HD may decrease the priority of seeking oral health care and maintaining good oral health [36].

Dental caries is caused in lieu of demineralization and remineralization of tooth structure that is continuously happening by interplay according to the pH of the biofilm [39]. Our study showed significantly lower mean DT and FT scores in cases than in controls. A similar finding has been obtained in various studies [16, 40]. It has been suggested that the caries activity in CKD patients on dialysis is lower because increased urea concentration in saliva leads to higher pH levels. Higher salivary urea could potentially protect teeth from demineralization. Andrade et al. [41] in their systematic review of six cross-sectional studies comparing DMFT of children and adolescents with CKD to healthy control have found lower caries experience among the cases. Menezes et al. [16] in their comparative cross-sectional study in Brazil noted no difference in caries experience between cases and controls. They, nevertheless, found a significant difference between mean filled teeth similar to our study.

Duration of dialysis did not seem to have a significant correlation with DMFT. DMFT index measures lifetime caries experience, it is possible that the effect of a relatively short duration of dialysis may not have been reflected on the DMFT index.

Worse periodontal health of the cases was seen to be associated with a longer duration of dialysis. However, the difference was not of statistical significance. Different studies have produced contrasting results regarding the influence of the duration of dialysis on periodontal health [42–44]. The use of a similar tool to measure periodontitis coupled with transparent reporting of the duration of dialysis may be helpful in comparison of findings and reaching a correct conclusion.

Another interesting finding was low self-reported gum bleeding but a high prevalence of periodontitis in cases. Typical periodontal disease is painless and it is not uncommon for periodontal disease to have reached a severe stage before its diagnosis [45].

In our study, the data on self-reported oral health suggested many of the participants (22.2%) had complaints of dry mouth. A similar finding was obtained by Dannewitz et al. [46] in Germany. Decreased salivary flow rate in patients with CKD on HD is well documented in previous studies [47–50]. Dry mouth is common in dialysis patients as a result of removal of excess fluids and medications that are used [51]. pH of saliva has also been shown to be increased in dialysis patients as compared to healthy controls [49].

Although only 9% of cases complained of oral lesions, the examiners observed oral ulcerations, and red and white patches in many of the cases. Similar lesions have been described to occur in patients receiving dialysis [52].

Similarly, 13% of the cases complained of tooth sensitivity to cold food and beverages. Many patients experience nausea and vomiting during dialysis. One study in Iran [53] has reported an incidence of vomiting of 11.7% among maintenance CKD cases under dialysis. Vomiting is known to cause erosion of teeth on the palatal aspect of maxillary anterior teeth. Similarly, increased loss of attachment was also seen in cases leading to root exposure and subsequently tooth sensitivity.

More cases (55%) reported having oral health problems but more than two-thirds of them had not made a single dental visit in their lifetime. A study by Singh et al. [54] in Dharan (near the hospital where our study was conducted) has reported 41% of individuals having made a dental visit which is more than cases (25%) but less than controls (68%) in our study. However, another community-based study done in a relatively remote place in eastern Nepal has reported a dental visit of 17% [55]. A smaller number of patients utilizing dental services in our study would also explain significantly less FT in cases as compared to healthy controls.

Brushing frequency among the cases was significantly different to that of controls. Infrequent brushing was common among the cases. The use of dental floss was extremely rare among our study participants (only two cases and two controls). Similar low use, albeit higher than our study, of dental floss, has been described in a study in central Nepal and China [56, 57]. The use of these oral hygiene measures has an impact on the periodontal and dental health of individuals.

Thirty-five percent of the cases and 20% among the controls had either been smoker at some point in their lifetime or currently smoke which is similar to that reported in a national survey [58].

Out of 54 cases, only one individual reported having received oral health education after starting dialysis. Considering the situation where 30 out of 54 felt that they had oral health problems, it looks unjustified. Adequate health education and appropriate treatment can help CKD cases better their oral health. If their oral health can be maintained at an optimal level, they could enjoy better oral health-related quality of life.

Our study, however, is not without limitations. It was a study conducted at a single center. Brushing frequency, pattern of dental visit, and tobacco usage was different between the groups.

Due to the cross-sectional design, it is not possible to establish the temporal antecedence of CKD on periodontitis or dental caries. Prospective studies on a larger CKD population would broaden the understanding of the oral health effects of CKD and the duration of dialysis on these parameters. Although variables like age and gender were matched, cases and controls were different regarding brushing frequency, dental visits, and tobacco usage, which can affect oral health. Smoking has been shown to have detrimental effect on the incidence and progression of periodontitis [59]. In addition, nonrandom sampling has been employed in the study, which may have compromised the external validity of the study for generalization. In light of this context, findings from our study should be interpreted with caution.

## 5. Conclusions and Recommendations

Oral health problems were found to be prevalent in CKD cases undergoing dialysis at a tertiary hospital in eastern Nepal. Dental visits are not sufficient to address those problems. All CKD cases should be made aware of the oral problems that exist and their potential effect on overall health. Oral hygiene measures that can be performed by patients themselves can be helpful in the prevention of these problems. Whenever possible, a multidisciplinary approach with the involvement of different specialties is recommended. Adequate communication between the treating physician, dentist, and patient is a sine qua non for efficient and evidence-based management of oral health problems in systemically ill patients.

Regional variations in oral health ask for strategies to be developed locally according to need. Despite the logistic challenges, HD services are becoming available to more CKD patients than before from all over Nepal. This means more CKD patients will survive for a longer time and will be able to enjoy a better quality of life. According to availability, HD for CKD patients is usually performed twice a week (for 3–4 hr at one visit) in Nepal. These patients spend a considerable amount of time in hospitals. Dental services are also being upgraded in different provinces of Nepal which means more dentists will be providing dental treatments to these patients. A provision of mandatory dental examination periodically (every 6 months) and provision of oral health instructions with necessary treatments can improve the oral health-related quality of life of these individuals. Policies should be changed and implemented according to the assessment of needs and evaluation of the situation. Common risk/health factors approach [60] should be considered to decrease the global health problems which are presenting serious challenges to developing countries like ours. This approach is helpful in the prevention of unnecessary duplication of efforts and compartmentalization of oral health.

This study shows an increased prevalence of periodontitis and a decreased prevalence of caries experience in CKD cases but it cannot establish causation. Population-based cohort studies with representative samples should be conducted to ascertain the unbiased effect of CKD on oral health. The results will be highly useful to the dental practitioners who are catering for the oral health needs of these cases. Moreover, studies regarding the oral health of mild to moderate kidney diseases should be a research priority and the oral health of such individuals should be taken care of.

## Figures and Tables

**Table 1:** Characteristics of the study participants from BPKIHS, September–November, 2020 (*n* = 104).

Parameter	Cases	Controls	Total (*n* = 108)	*p*-Value
Age (mean ± SD)	47 ± 12.41	46.78 ± 12.13	47.09 ± 12.23	0.790^#^
Gender, *n* (%)				
Male	26 (48.1%)	26 (48.1%)	52	1.000^ǂ^
Female	28 (51.9%)	28 (51.9%)	56	
Education, *n* (%)				
Illiterate	13 (24.1%)	8 (14.8%)	21	0.087 ^*∗*^
Primary education	27 (50%)	21 (38.9%)	48	
Secondary or higher	14 (25.9%)	25 (46.3%)	39	
Frequency of tooth brushing, *n* (%)				
Once a day	34 (63%)	34 (63%)	68	**0.036** ^ǂ^
Twice a day	8 (14.8%)	16 (29.6%)	24	
Sometimes	12 (22.2%)	4 (7.4%)	16	
Use of dental floss, *n* (%)				
Yes	2 (3.7%)	2 (3.7%)	4	0.938** ^*∗*^**
No	20 (376%)	18 (35.2%)	38	
Don't know	32 (59.3%)	34 (63%)	66	
Dental visit, *n* (%)				
Within 6 months	2 (3.7%)	15 (27.8%)	17	**<0.001** ^ǂ^
Before 6 months	13 (24.1%)	22 (40.7%)	35	
Never	39 (72.2%)	17 (31.5%)	56	
Tobacco usage, n (%)				
Never smoker	35 (64.8%)	43 (79.6%)	78	**<0.001 ^*∗*^**
Previous smoker	19 (35.2%)	4 (7.4%)	23	
Current smoker	0 (0%)	7 (13%)	7	

^#^Independent sample T test. ^ǂ^*χ*^2^ test.  ^*∗*^Fishers exact test. Bold signifies statistically significant difference.

**Table 2:** Oral health status of study population from BPKIHS, September–November, 2020 (*n* = 104).

Parameter mean ± SD (median, IQR)	Case	Control	Overall	*p*-Value
Decayed teeth	0.48 ± 0.74	0.91 ± 1.01	0.69 ± 0.91	**0.013 ^*∗*^**
	(0,1)	(1,1)	(0,1)	
Missing teeth	2.35 ± 4.02	2.17 ± 2.26	2.26 ± 3.38	0.572 ^*∗*^
	(1.5,3)	(2,2)	(1.5,3)	
Filled teeth	0.11 ± 0.60	0.65 ± 1.32	0.38 ± 1.05	**<0.001 ^*∗*^**
	(0,0)	(0,1)	(0,0)	
Decayed missing filled teeth	2.91 ± 4.04	3.72 ± 2.99	3.31 ± 3.56	**0.013 ^*∗*^**
	(3,3)	(3,3)	(3, 3)	
Decayed surfaces	0.50 ± 0.86	1.22 ± 1.42	0.86 ± 1.22	**0.001 ^*∗*^**
	(0,2)	(1,2)	(0,2)	
Missing surfaces	11.69 ± 19.48	10.74 ± 13.02	11.21 ± 16.50	0.638 ^*∗*^
	(7,15)	(9.5, 10)	(7,15)	
Filled surfaces	0.15 ± 1.08	0.98 ± 1.94	0.56 ± 1.62	**<0.001 ^*∗*^**
	(0,0)	(0, 1)	(0,0)	
Decayed missing filled surfaces	12.33 ± 19.43	12.94 ± 13.30	12.64 ± 16.58	0.149 ^*∗*^
	(10,10)	(10,10)	(10,10)	
Periodontitis, *n* (%)				
None	6 (11.1%)	19 (35.2%)	25 (23.1%)	**<0.001ᶧ**
Mild	11 (20.4%)	19 (35.2%)	30 (27.8%)	
Moderate	24 (44.4%)	14 (25.9%)	38 (35.2%)	
Severe	13 (24.1%)	2 (3.7%)	15 (13.9%)	

^*∗*^Mann–Whitney *U* test. ᶧ*χ*^2^ test, Bold signifies statistically significant difference.

## Data Availability

The data that support the findings of this study are available from the corresponding author upon reasonable request.
